# Subdominant/Cryptic CD8 T Cell Epitopes Contribute to Resistance against Experimental Infection with a Human Protozoan Parasite

**DOI:** 10.1371/journal.pone.0022011

**Published:** 2011-07-14

**Authors:** Mariana R. Dominguez, Eduardo L. V. Silveira, José Ronnie C. de Vasconcelos, Bruna C. G. de Alencar, Alexandre V. Machado, Oscar Bruna-Romero, Ricardo T. Gazzinelli, Mauricio M. Rodrigues

**Affiliations:** 1 Centro de Terapia Celular e Molecular (CTCMol), Universidade Federal de São Paulo-Escola Paulista de Medicina, São Paulo, Brazil; 2 Departamento de Microbiologia, Imunologia e Parasitologia, Universidade Federal de São Paulo-Escola Paulista de Medicina, São Paulo, Brazil; 3 Centro de Pesquisas René Rachou, FIOCRUZ, Belo Horizonte, Minas Gerais, Brazil; 4 Departamento de Microbiologia, Instituto de Ciências Biológicas, Universidade Federal de Minas Gerais, Belo Horizonte, Minas Gerais, Brazil; 5 Departamento de Bioquímica e Imunologia, Universidade Federal de Minas Gerais, Belo Horizonte, Minas Gerais, Brazil; 6 Division of Infectious Disease and Immunology, Department of Medicine, University of Massachusetts Medical School, Worcester, Massachusetts, United States of America; Université Pierre et Marie Curie, FRANCE

## Abstract

During adaptive immune response, pathogen-specific CD8^+^ T cells recognize preferentially a small number of epitopes, a phenomenon known as immunodominance. Its biological implications during natural or vaccine-induced immune responses are still unclear. Earlier, we have shown that during experimental infection, the human intracellular pathogen *Trypanosoma cruzi* restricts the repertoire of CD8^+^ T cells generating strong immunodominance. We hypothesized that this phenomenon could be a mechanism used by the parasite to reduce the breath and magnitude of the immune response, favoring parasitism, and thus that artificially broadening the T cell repertoire could favor the host. Here, we confirmed our previous observation by showing that CD8^+^ T cells of H-2^a^ infected mice recognized a single epitope of an immunodominant antigen of the *trans*-sialidase super-family. In sharp contrast, CD8^+^ T cells from mice immunized with recombinant genetic vaccines (plasmid DNA and adenovirus) expressing this same *T. cruzi* antigen recognized, in addition to the immunodominant epitope, two other subdominant epitopes. This unexpected observation allowed us to test the protective role of the immune response to subdominant epitopes. This was accomplished by genetic vaccination of mice with mutated genes that did not express a functional immunodominant epitope. We found that these mice developed immune responses directed solely to the subdominant/cryptic CD8 T cell epitopes and a significant degree of protective immunity against infection mediated by CD8^+^ T cells. We concluded that artificially broadening the T cell repertoire contributes to host resistance against infection, a finding that has implications for the host-parasite relationship and vaccine development.

## Introduction

MHC class Ia-restricted CD8^+^ T cells are important mediators of the adaptive immune response against infections caused by intracellular microorganisms, including the digenetic intracellular protozoan parasite *Trypanosoma cruzi*, the causative agent of Chagas disease (American trypanosomiasis). During experimental infection, this T cell subpopulation has been shown to be critical for host survival even when small doses of parasites are used in challenges [1]–[5]. In spite of the CD8^+^ T-cell mediated immune response, the parasite survives within the host and establishes a life-long chronic infection. Parasite persistence is considered one of the critical factors in the development of the complex immunopathology caused by *T. cruzi* that may occur years after the initial infection in ∼30% of infected individuals [6]–[11]. Thus, understanding how the parasites escape the immune response and persist for such long periods may help us to find new means for interventions against Chagaś disease that would improve quality of life for millions of infected individuals in Latin America

Recent studies on the CD8^+^ T-cell immune responses that occur during experimental *T. cruzi* infection in inbred mouse strains described a surprising immunodominance of certain epitopes expressed by members of a large family of *T. cruzi* surface antigens named *trans*-sialidases (TS) [1], [5], [12]–[20]. How and why this strong pattern of immunodominance is established is still a matter of debate. In general terms, immunodominance can emerge as a result of different mechanisms that regulate the formation of the complex of MHC-I-peptide on the surface of antigen presenting cells (APC) such as antigen concentration, stability or epitope availability after processing and translocation to the endoplasmic reticulum, where the MHC-I-peptide complex is assembled to be transported to the APC surface [21]–[24].

After a stable MHC-I-peptide complex is formed on the surface of the APC, factors related to CD8^+^ T cells, such as the frequency of precursors, their TCR affinities, their capacity to proliferate in response to antigen and, thus, be incorporated into the pool of responder cells, are factors that shape immunodominance hierarchies. These factors transcend the MHC restriction element and may create T-cell competition for APCs and other resources, enabling certain CD8^+^ T cells to dominate and suppress others [23]–[29].

By comparing the specificity of CD8^+^ T cells of homozygous and heterozygous mouse strains, we observed that the immunodominance that occurs during experimental *T. cruzi* infection could be exerted not only on epitopes restricted by the same MHC molecules but also, unexpectedly, on the immune response to epitopes restricted by different MHC-I molecules. This phenomenon, termed cross-competition, represents a potent means by which T cells with a certain specificity may become immunodominant [30]–[34]. This strong and unusual phenomenon has been shown to be due to *T. cruzi* infection because following immunization with recombinant adenovirus expressing the same parasite antigens, this pattern of immunodominance was not observed [15].

Based on these observations, we hypothesized that this competition/immunodomination between T cells of different specificities could be a sophisticated strategy that *T. cruzi* developed to reduce the breath and magnitude of CD8^+^ T-cell responses, suppressing the immune responses of these T cells with other specificities in order to escape complete elimination by host effector cells. Thus, we expected that artificially broadening the immune response to include T cells specific for subdominant or cryptic epitopes could favor the host, counteracting the restriction imposed by the infection. Here, we tested this hypothesis by using mice genetically immunized with a mutated form of the *amastigote surface protein (asp) - 2* gene in which the immunodominant CD8 T cell epitope is no longer functional. The CD8 T cell-mediated immune response of these mice was directed only to the newly described subdominant/cryptic CD8 T cell epitopes of ASP-2. Even in the absence of an immune response directed to the immunodominant epitope, these mice displayed a significant degree of protective immunity, albeit not as strong as the immune response elicited by the original gene expressing both the immunodominant and the subdominants epitopes. These results are compatible with our hypothesis that artificially broadening the immune response favors the host. Indirectly, we suggest that immunodominance may in fact be a mechanism to establish a chronic infection.

## Materials and Methods

### Ethics Statement

All experimental procedures were approved by the Ethics Committee for Animal Care of the Federal University of São Paulo (Id # CEP 0426/09).

### Mice and parasites

Female 8-week-old H-2^a^ mice (B10.A and A/Sn) were purchased from CEDEME (Federal University of São Paulo). Bloodstream trypomastigotes of the Y strain of *T. cruzi* were obtained from A/Sn mice infected 7–8 days earlier [12]. Each B10.A or A/Sn mouse was challenged i.p. with a final dose containing 10^4^ or 150 parasites, respectively, in a final volume of 0.2 mL. Parasite development was monitored by counting the number of bloodstream trypomastigotes in 5 µL of fresh blood collected from the tail vein [12].

### Peptides

Peptides were purchased from Genscript (Piscataway, NJ). Purity was as follows: TEWETGQI (95%); PETLGHEI (97.4%); YEIVAGYI (99.40%); TPTAGLVGF (98.6%); GSRNGNDRL (97.1%); ESKSGDAPL (96.1%); HEHNLFGI (98.7%); ESSTPTAGL (99.1%); ESEPKRPNM (98.7%); VSWGEPKSL (99.2%); YSDGALHLL (97.3%); AESWPSIV (96.5%); and RPNMSRHLF (99.4%).

### Recombinant plasmids and adenoviruses

Plasmid pIgSPCl.9 and the human replication-defective adenovirus type 5 containing the *asp-2* gene were obtained as described previously [35], [36]. Mutated *asp-2* was generated by a series of PCR reactions using DNA encoding the *asp-2 clone 9* gene as a template (Genbank Accession Number: AY186572). In the first reaction, the forward and reverse oligonucleotides were as follows:

- 5′-GGGGGTACCATGCTCTCACGTGTTGCT-3′;
- 5′-GAACGATCATGAGTGCTTGGCCCGTCTCCCATGCGGTGATGCGGGGATC-3′

In the second reaction, they were as follows:

- 5′-GATCCCCGCATCACCGCATGGGAGACGGGACAAGCACTCATGATCGTTC-3′
- 5′-GGGTCTAGATCAGACCATTTTTAGTTCACC-3′.

PCR products were purified, mixed and subjected to a third PCR reaction. Forward and reverse oligonucleotides were respectively

- 5′-GGGGGTACCATGCTCTCACGTGTTGCT-3′;
- 5′-GGGTCTAGATCAGACCATTTTTAGTTCACC-3′.

The final PCR product was completely sequenced. The only modifications found were in the nucleotide sequences encoding the immunodominant epitope TEWETGQI. The new sequence encoded the amino acids (AA) TAWETGQA. This plasmid is referred to as pIgSpTAWETGQA. The new gene was also subcloned into the pAdCMV shuttle vector, and the recombinant replication-defective adenovirus human adenovirus 5 was produced by Vectors BioLabs, Philadelphia, USA. This new recombinant adenovirus is referred to as AdTAWETGQA. Viruses and plasmids were purified as described previously [35]–[37]. Mice were inoculated intra-muscularly (i.m.) in each *tibialis anterioris* muscle with 50 µg of plasmid DNA 3 times every 3 weeks.

Heterologous prime-boost immunization consisted of priming i.m. with a total of 100 µg of plasmid DNA followed by a dose of viral suspension containing 2×10^8^ plaque forming units (pfu) of adenovirus twenty-one days later in the same locations. Immunological assays or challenges were performed 14 days after viral inoculation.

*In vivo* depletion of CD8^+^ T cells were performed by treating vaccinated A/Sn mice with 53.6.7 MAb. At days 2 and 3 before challenge with trypomastigotes, mice were injected i.p. with a dose of 1 mg of anti-CD8 or control Rat IgG. Seven days after challenge, each mouse received one more dose of 1 mg of anti-CD8 or Rat IgG. The efficacy of depletion of CD8^+^ spleen cells before challenge was more than 95% in anti-CD8 treated mice compared to Rat IgG treated ones.

### Immunological T cell assays

*Ex vivo* ELISPOT (IFN-γ) or *in vivo* cytotoxic assays were performed exactly as described previously [12], [15]. The surface mobilization of CD107a and the intracellular expression of cytokines (IFN-γ, TNF-α, IL-2 and IL-10) was evaluated after *in vitro* culture of splenocytes in the presence or absence of antigenic stimulus. Cells were washed three times in plain RPMI and re-suspended in cell culture medium consisting of RPMI 1640 medium, pH 7.4, supplemented with 10 mM Hepes, 0.2% sodium bicarbonate, 59 mg/l of penicillin, 133 mg/l of streptomycin, and 10% Hyclone fetal bovine sera (Hyclone, Logan, Utah). The viability of the cells was evaluated using 0.2% Trypan Blue exclusion dye to discriminate between live and dead cells. Cell concentration was adjusted to 5×10^6^ cells/mL in cell culture medium containing anti-CD28 (2 µg/mL), Brefeldin A (10 µg/mL), Monensin (5 µg/mL) and FITC-labeled anti-CD107a (Clone 1D4B, 2 µg/mL, BD Pharmingen). In half of the cultures, a final concentration of 10 µM of the VNHRFTLV peptide was added. The cells were cultivated in flat-bottom 96-well plates (Corning) in a final volume of 200 µl in duplicate, at 37°C in a humid environment. After a 20-h incubation, cells were stained for surface markers with Per-CP or PE-labeled anti-CD8 on ice for 20 min. To detect IFN-γ, TNF-α? IL-2 and IL-10 by intra-cellular staining (ICS), cells were then washed twice in buffer containing PBS, 0.5% BSA and 2 mM EDTA, fixed in 4% PBS-paraformaldehyde solution for 10 minutes and permeabilized for 15 minutes in a PBS, 0.1% BSA, 0.1% saponin solution. After being washed twice, cells were stained for intracellular markers using APC or PE-labeled anti-IFN-γ (Clone XMG1.2) and? PE- labeled anti-TNF-α (clone MP6-XT22), APC-labeled anti-IL-2 (clone JES6-5H4) or APC-labeled anti-IL-10 (JES5-16E3) for 20 minutes on ice. Finally, cells were washed twice and fixed in 1% PBS-paraformaldehyde. At least 300,000 cells were acquired on a BD FacsCanto flow cytometer and then analyzed with FlowJo.

### Statistical analysis

Values were expressed as means ± SD. These values were compared using one-way ANOVA followed by Tukey's HSD tests (http://faculty.vassar.edu/lowry/VassarStats.html). The LogRank test was used to compare mouse survival rates after challenge with *T. cruzi* (http://bioinf.wehi.edu.au/software/russell/logrank/). The differences were considered significant when the *P* value was <0.05.

## Results

**Figure 1:**
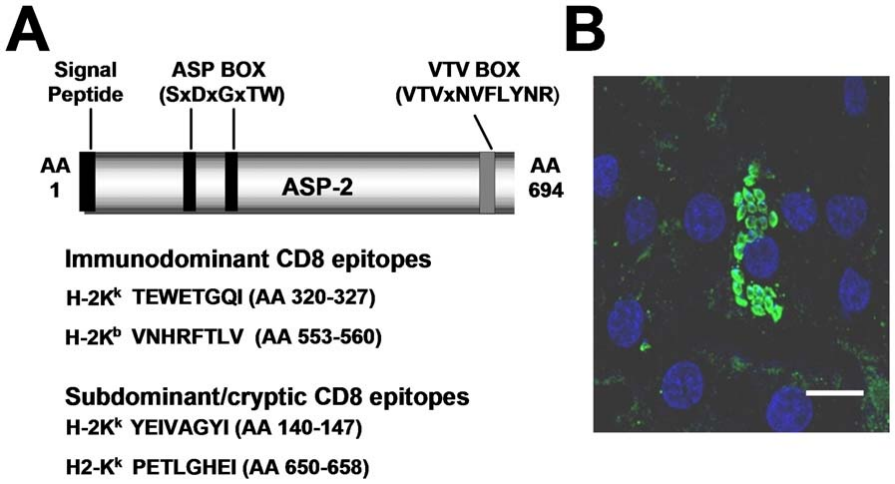
Structure and localization of Amastigote Surface Protein-2 (ASP-2). A- Schematic view of the primary structure of *T. cruzi* ASP-2. B- HeLa cells were infected for 48 h with trypomastigotes of the Y strain. After fixation, indirect immunofluorescence or DAPI staining were performed as described using MAb K22 and imaged using fluorescence microscopy [39]. Bar, 14 µM.

During experimental infection of H-2^b^ or H-2^a^ inbred mouse strains with parasites of the Y strain of *T. cruzi*, two epitopes were identified within the ASP-2 antigen represented by the VNHRFTLV or TEWETGQI peptides. They were recognized by H-2K^b^- or H-2K^k^-restricted CD8^+^ cytotoxic T cells, respectively [12], [14], [15], [37], [38]. ASP-2 is a member of the large family of TS surface antigens and is abundantly expressed only in the intracellular forms (amastigotes) of *T. cruzi* [39]. Fig. 1A and Fig. 1B show, respectively, some of the structural features of ASP-2 antigen and its expression by amastigotes.

**Figure 2:**
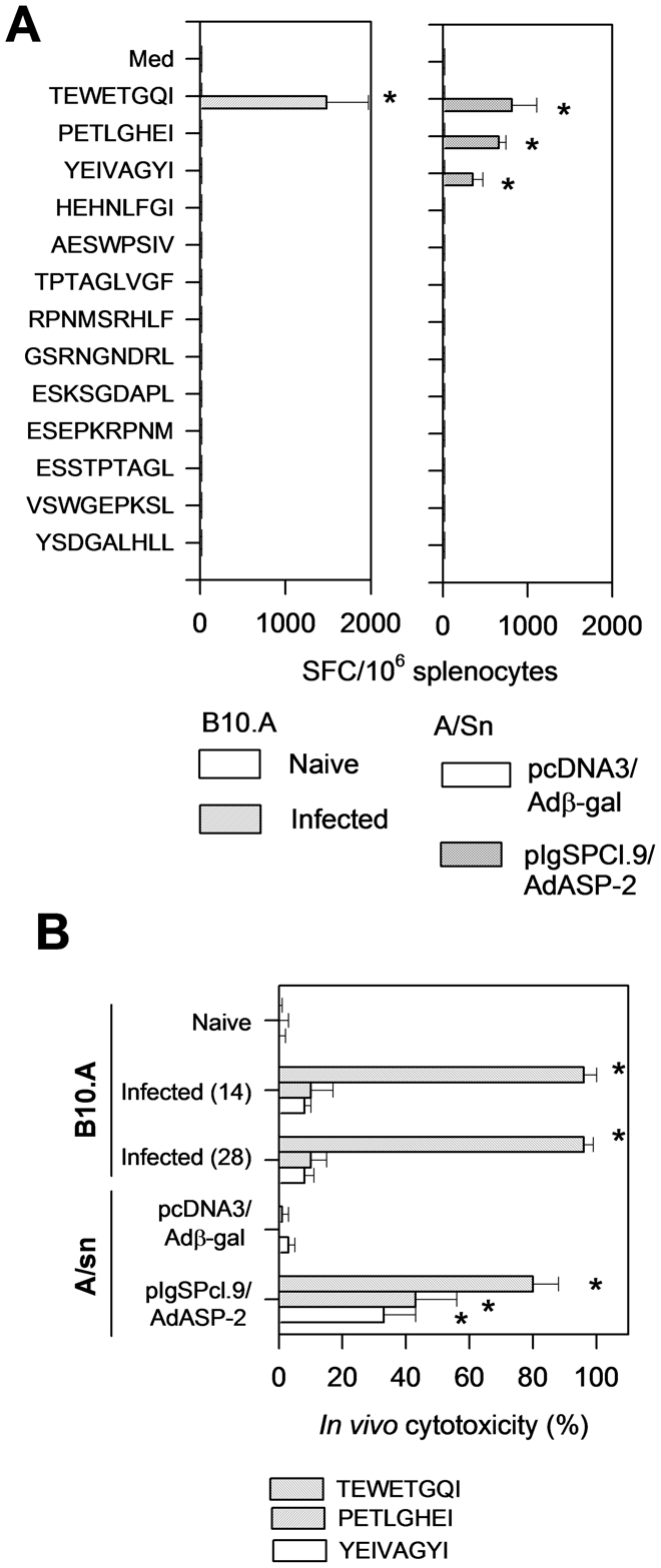
CD8 T-cell epitope identification during immune responses of H-2^a^ mice infected with *T. cruzi* or genetically vaccinated with *asp-2* gene. B10.A mice were infected with 10^4^ *T. cruzi* blood parasites. A/S mice were immunized with pIgSPCl.9 followed by AdASP-2 and injected i.m. at 0 and 3 weeks, respectively. Control mice were naïve or injected with pcDNA3 followed by pcDNA3/Adβ-gal. **A**- Two weeks after infection or the final immunizing dose, splenic cells were re-stimulated *in vitro* in the presence of medium or the indicated peptides at a final concentration of 10 µM. The number of splenic IFN-γ spot-forming cells (SFC) was estimated by *ex vivo* ELISPOT assay. **B**- *In vivo* cytotoxic activity was estimated by injecting each mouse with syngeneic CFSE-labeled splenic cells coated with or without 2 µM of the indicated peptide. Results are expressed as means ± SD of 4 mice per group and are representative of experiments performed at least twice with similar results. Asterisks denote that the number of SFC or *in vivo* cytotoxicity were significantly higher when compared to naïve or pcDNA3/Adβ-gal injected mice (P<0.01, one-way ANOVA).

To test whether the H-2K^k^-restricted epitope TEWETGQI could be an immunodominant epitope, a series of synthetic peptides containing the predicted AA anchor motif for binding to the H-2K^k^ or H-2L^d^ alleles of mouse MHC haplotype H-2^a^ was employed. No epitope was predicted to bind to H-2D^d^ (Table 1). After infection of H-2^a^ mice, antigen-specific IFN-γ producing cells could only be detected in the presence of the peptide TEWETGQI (Fig. 2A). We concluded that this epitope was the immunodominant epitope of ASP-2 during infection of H-2^a^ mice with the Y strain of *T. cruzi*. H-2^a^ mice used in this experiment were B10.A because they are resistant to infection with *T. cruzi*.

**Table 1:** Peptides used in the study.

Peptide	AA positions	Predicted H-2 restriction
T**E**WETGQ**I**	320–327	K^k^
P**E**TLGHE**I**	650–657	K^k^
Y**E**IVAGY**I**	140–147	K^k^
H**E**HNLFG**I**	130–137	K^k^
A**E**SWPSI**V**	121–128	K^k^
T**P**TAGLVG**F**	488–496	L^d^
R**P**NMSRHL**F**	36–44	L^d^
G**S**RNGNDR**L**	172–179	L^d^
E**S**KSGDAP**L**	69–77	L^d^
E**S**EPKRPN**M**	31–39	L^d^
E**S**STPTAG**L**	485–493	L^d^
V**S**WGEPKS**L**	239–247	L^d^
Y**S**DGALHL**L**	438–446	L^d^

Peptides were selected by the scores determined by programs available at the sites: http://www.syfpeithi.de/and http://www-bimas.cit.nih.gov/molbio/hla_bind/.

Putative anchor residues are in bold and underlined.

We showed previously that the strong pattern of immunodominance observed following infection with *T. cruzi* was not duplicated in mice genetically immunized with a recombinant adenovirus expressing ASP-2 (AdASP-2, ref. 15). Then, we determined whether genetically immunized H-2^a^ mice could present a different pattern of immunodominance. We used a genetic immunization approach which consisted of a heterologous prime-boost regimen using plasmid DNA followed by a recombinant adenovirus both containing the same *asp-2* gene. This protocol provided strong and long lasting protective immunity against experimental infection mediated by CD8^+^ T cells [37], [38]. In these experiments, we used H-2^a^ mice of the A/Sn strain. These mice are highly susceptible to infection with *T. cruzi*, allowing us to perform protective immunity studies [37], [38]. Nevertheless, it is important to mention that the results were similar when we used B10.A mice.

After *ex vivo* stimulation with our synthetic peptides, as expected, IFN-γ producing cells were detected following stimulation with the TEWETGQI peptide. In addition to this epitope, two other epitopes (PETLGHEI and YEIVAGYI) induced IFN-γ production by immune cells (Fig. 2A). These peptide-specific IFN-γ producing cells were CD8^+^ T cells as determined by simultaneous staining of intra-cellular IFN-γ and the surface marker CD8 (see below).

These peptides were recognized by cytotoxic cells in H-2^a^ mice as determined by *in vivo* cytotoxicity assays using target cells coated with each of these peptides. B10.A mice infected with *T. cruzi* developed strong *in vivo* cytotoxicity against target cells coated with the peptide TEWETGQI. In contrast, very limited (if any) *in vivo* cytotoxicity was observed against target cells coated with peptides PETLGHEI and YEIVAGYI. These results were not due to different kinetics of the immune response because we observed the same results 14 or 28 days after an infectious challenge (Fig. 2B).

However, A/Sn (H-2^a^) mice genetically vaccinated with a heterologous prime-boost vaccination regimen displayed easily detectable *in vivo* cytotoxic activity against target cells coated with any of these three peptides. The elimination of target cells coated with peptide TEWETGQI was always stronger than the two others, suggesting a pattern of immunodominance.

**Figure 3:**
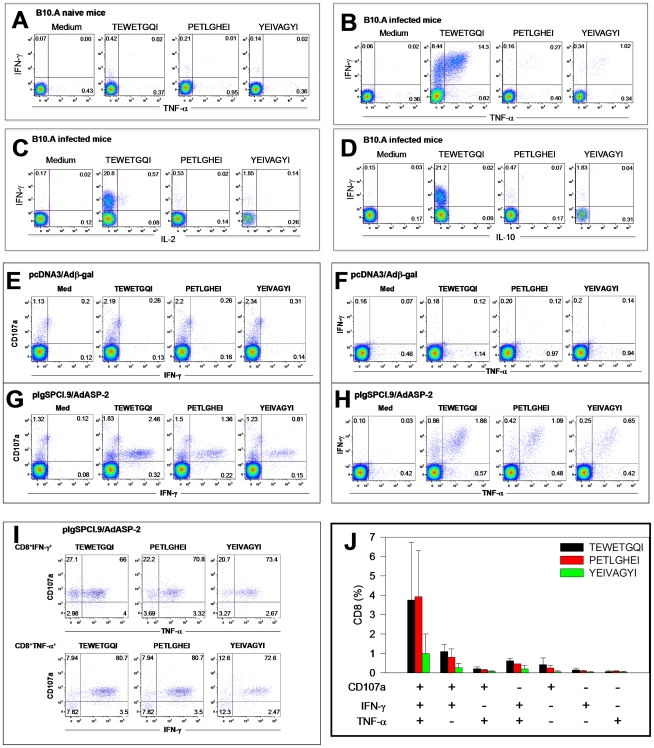
Surface mobilization of CD107a and expression of IFN-γ, TNF-α, IL-2 or IL-10 by specific CD8^+^ T cells from B10.A mice infected with *T. cruzi* or A/Sn mice immunized with pIgSCl.9/AdASP-2 vaccine. B10.A or A/Sn mice were infected or immunized as described in the legend of Fig. 2. Control mice were either naive mice or mice immunized with pCDNA3/Adβ-gal. Twenty one or fourteen days after infection or immunization, respectively, these mice had their splenic cells cultured in the presence of anti-CD107a and anti-CD28, with or without the peptides TEWETGQI, PETLGHEI or YEIVAGYI. After 12 h, cells were stained for CD8, IFN-γ, TNF-α, IL-2 and IL-10. Representative analyses (medians) are shown from four mice performed per experiment. **A**) Example of splenic CD8^+^ cells from B10.A naive mice cultivated *in vitro* in the presence of medium alone (Medium) or with the indicated peptides and stained for expression of IFN-γ and TNF-α. **B, C and D**) Examples of splenic CD8^+^ cells from B10.A infected mice cultivated *in vitro* in the presence of medium alone (Medium) or with the indicated peptides and stained for expression of: B) IFN-γ and TNF-α; C) IFN-γ and IL-2; D) IFN-γ and IL-10. **E and F**) Examples of splenic CD8^+^ cells from mice immunized with pcDNA3/Adβ-gal cultivated *in vitro* in the presence of medium alone (Medium) or with the indicated peptides and stained for surface mobilization of CD107a and expression IFN-γ (panel E) or expression of IFN-γ and TNF-α (panel F). **G and H**) Examples of splenic CD8^+^ cells from mice immunized with pIgSPCl.9/AdASP-2 cultivated *in vitro* in the presence of medium alone (Medium) or with the indicated peptides and stained for surface mobilization of CD107a and expression IFN-γ (panel G) and expression IFN-γ and TNF-α (panel H). **I and J**) Determination of multifunctional CD8^+^ cells from mice immunized with pIgSPCl.9/AdASP-2 cultivated *in vitro* in the presence of the indicated peptides and stained for surface mobilization of CD107a and expression IFN-γ and TNF-α.

To determine whether other cytokines and/or effector molecules could be secreted by peptide-specific T cells, we performed staining to detect surface mobilization of CD107a (a marker for exocytosis) or intra-cellular accumulation of IFN-γ, TNF-α, IL-2 or IL-10. When we used splenic cells from B10.A mice infected with *T. cruzi*, we observed that upon stimulation with TEWETGQI, a large fraction of CD8^+^ cells mobilize CD107a to the surface (data not shown) and accumulate intra-cellular IFN-γ and TNF-α (Fig. 3B). These CD8^+^ cells were therefore multifunctional CD107a^+^IFN-γ^+^TNF-α^+^ (∼35%) or IFN-γ^+^TNF-α^+^ (∼40%). We were unable to detect the presence of significant numbers of IL-2 or IL-10 expressing cells in these same samples (Fig. 3C and 3D). The expression of these cytokines was dependent on the infection because they were not detected in cells from naive mice (Fig. 3A).

In contrast, a relatively low frequency of CD8^+^ splenic cells stimulated with peptides PETLGHEI or YEIVAGYI mobilized CD107a to the surface (data not shown) or accumulated intra-cellular IFN-γ or TNF-α (Fig. 3B). We were also unable to detect the presence of significant numbers of IL-2 or IL-10 expressing cells in these same cells (Fig. 3C and 3D).

Splenic cells from A/Sn mice genetically vaccinated with heterologous prime-boost regimen were stimulated with peptides TEWETGQI, PETLGHEI or YEIVAGYI. The results shows that a large fraction of the CD8^+^ cells of pIgSPCl.9/AdASP-2 immunized mice at the same time mobilize CD107a to the surface and expressed intra-cellular IFN-γ and TNF-α (Fig. 3G to 3J). These cells were therefore multifunctional CD8^+^ T cells as we have previously described [37]. We were unable to detect the presence of intra-cellular IL-2 or IL-10 in these same cells (data not shown). These results confirmed and extended the ones described in Fig. 2).

The description of these two new epitopes allowed us to test whether immunity to the subdominant/cryptic epitope could participate during protective immunity against *T. cruzi* infection, a phenomenon that has not previously been tested experimentally. For this purpose, we generated a plasmid DNA and a recombinant adenovirus containing a mutated form of the *asp-2* gene in which we modified the nucleotides encoding the anchor residues required for the immunodominant TEWETGQI epitope to bind to the H-2K^k^ molecule. The mutated gene expressed the AA sequence TAWETGQA, where the alanines (A) replaced glutamic acid (E) or isoleucine (I) of the original epitope. In preliminary experiments, we observed that the synthetic peptide TAWETGQA was not recognized by immune cells from genetically vaccinated H-2^a^ mice (data not shown). Details of the plasmid and recombinant adenovirus containing the mutated form of the *asp-2* gene are shown in Table 2.

**Table 2:** Genetic vectors used in the study.

Designation	Vector	ASP-2 CD8 epitopes		
pIgSPCl.9	Plasmid	TEWETGQI	PETLGHEI	YEIVAGYI
pIgSPTAWETGQA.9	Plasmid	TAWETGQA	PETLGHEI	YEIVAGYI
AdASP-2	Adenovirus	TEWETGQI	PETLGHEI	YEIVAGYI
AdTAWETGQA	Adenovirus	TAWETGQA	PETLGHEI	YEIVAGYI

**Figure 4:**
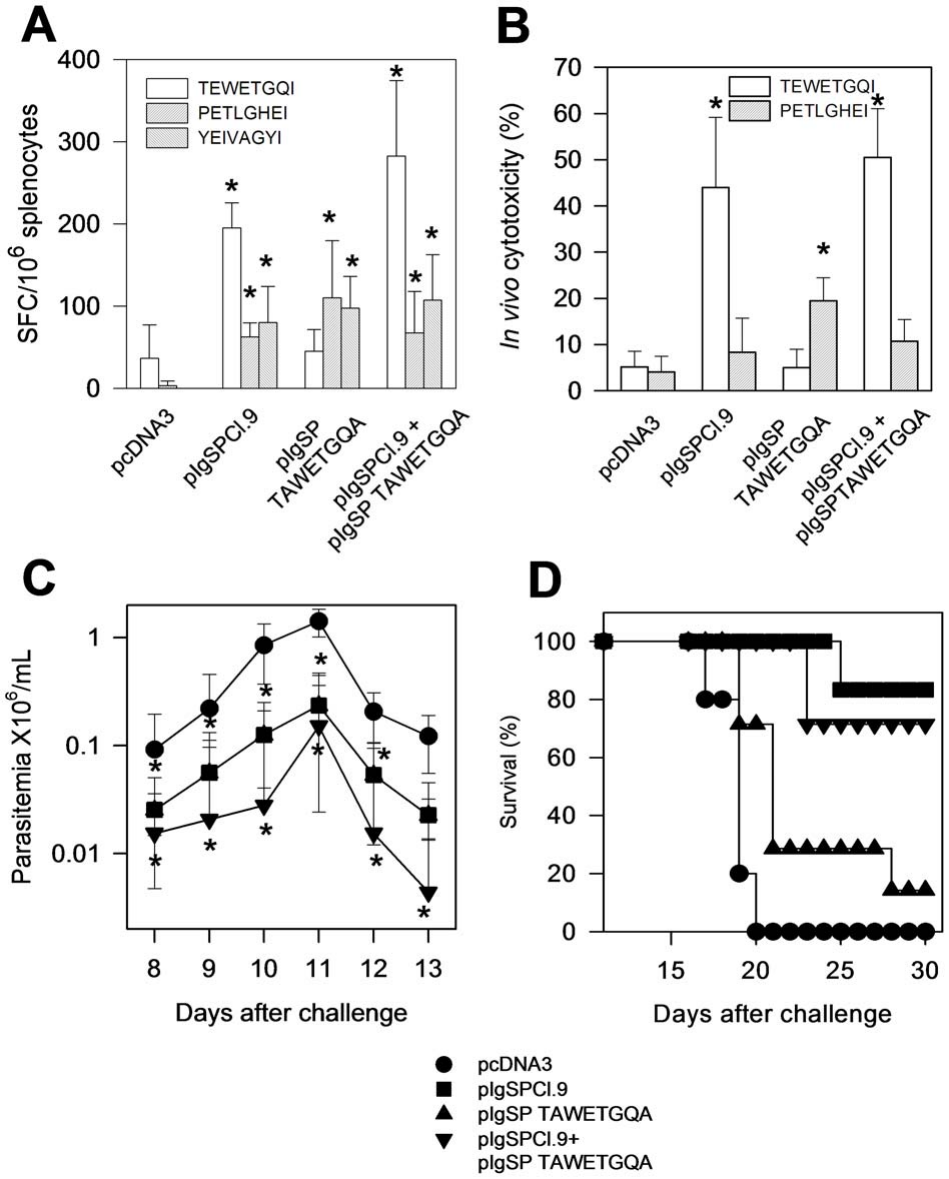
CD8 immune responses and trypomastigote-induced parasitemia and mortality in A/Sn mice genetically immunized with different plasmid DNA containing the *asp-2* gene. A/Sn mice were immunized with pcDNA3, pIgSPCl.9, pIgSPTAWETGQA or simultaneously with the last two (pIgSPCl.9 and pIgSPTAWETGQA). Immunization consisted of 3 doses of 100 µg of DNA each given by the i.m. route in the *tibialis anterioris* three weeks apart. A- Two weeks after the final immunizing dose, mice were challenged i.p. with 150 bloodstream trypomastigotes. Two weeks after infection, splenic cells were re-stimulated *in vitro* in the presence of medium only or the indicated peptides at a final concentration of 10 µM. The number of splenic IFN-γ spot-forming cells (SFC) was estimated by *ex vivo* ELISPOT assay. B- *In vivo* cytotoxic activity was estimated by injecting each mouse with syngeneic CFSE-labeled splenic cells coated with or without 2 µM of the indicated peptide. Results are expressed as means ± SD of 4 mice per group and are representative of experiments performed at least twice with similar results. Asterisks denote that the number of SFC, or the *in vivo* cytotoxicity, were significantly higher when compared to SFC found in naïve or pcDNA3/Adβ-gal-injected mice (*P*<0.01). C- Parasitemia for each mouse group is represented as mean ± SD (n = 5–7). Asterisks denote that mice from groups immunized with pIgSPCl.9 or pIgSPTAWETGQA or both had significantly lower parasitemia (*P*<0.01) than animals injected with pcDNA3. The curves of parasitemia of animals immunized with pIgSPCl.9 (squares) or pIgSPTAWETGQA (triangles) are superimposed. D- Kaplan-Meier curves for the survival of mouse groups immunized and challenged as described above (n = 5–7). Mice from groups immunized with pIgSPCl.9 or pIgSPCl.9/pIgSPTAWETGQA survived significantly longer than animals injected with pcDNA3 or pIgSPTAWETGQA (P<0.05, in all cases, LogRank test). Mice immunized with pIgSPTAWETGQA also survived longer than animals injected with pcDNA3 (*P*<0.05). No animals died after the 30^th^ day until they were euthanized. Results are representative of two independent experiments.

Initially, we genetically immunized mice with plasmids containing the original gene (pIgSPCl.9), the mutated gene (pIgSPTAWETGQA) or both plasmids simultaneously. Immune responses were estimated by ELISPOT after *ex vivo* stimulation with the immunodominant epitope TEWETGQI or with the subdominant/cryptic epitopes PETLGHEI or YEIVAGYI two weeks after challenge with *T. cruzi*. We chose this protocol because, as the immune response following plasmid DNA vaccination is usually low, it is easier to detect the anamnestic immune responses after challenge [12]. We observed that all mice immunized with *asp-2* genes (mutated or not) presented specific IFN-γ producing cells when stimulated with subdominant/cryptic epitopes (PETLGHEI or YEIVAGYI, Fig. 4A). The immune response was specific because mice immunized with control plasmid pcDNA3 failed to recognize these peptides. However, we detected IFN-γ producing cells specific to TEWETGQI only in mice immunized with plasmid pIgSPCl.9. It is noteworthy that the number of cells detected in mice immunized with pIgSPTAWETGQA was similar to the number of cells in pcDNA3 injected mice (Fig. 4A). These numbers reflect cells primed during infection. Analysis of *in vivo* cytotoxic activity also demonstrated that in mice immunized with the plasmid pIgSPTAWETGQA, the response to the immunodominant epitope TEWETGQI was not different from control mice immunized with pcDNA3 (Fig. 4B). These immunological analyses demonstrated that mice immunized with pIgSPTAWETGQA indeed lost the functional immunodominant TEWETGQI response but had an unaltered ability to elicit immune responses to the subdominant epitopes PETLGHEI and YEIVAGYI.

After challenge, mice immunized with plasmids containing the *asp-2* gene (mutated or not) presented significantly lower parasitemia than control mice immunized with pcDNA3 (Fig. 4C). Although the levels of parasitemia were not statistically different when compared to mice vaccinated with the plasmid containing the mutated *asp-2* gene, the mortality of these animals was significantly faster (Fig. 4D). We therefore concluded that broadening the CD8^+^ T cell immune response by vaccination with a plasmid containing epitopes that elicit immune responses to subdominant/cryptic epitopes of ASP-2 could provide some degree of protective immunity. Nevertheless, because protective immunity elicited by vaccination with pIgSPTAWETGQA was not as efficient, the presence of a functional immunodominant epitope was clearly important for effective protective immunity.

We then sought to test the same hypotheses described above using a distinct approach. For that purpose, H-2^a^ mice were primed with plasmid pIgSPCl.9 followed by a booster immunization with AdASP-2 (heterologous prime- boost regimen). Alternatively, mice were primed with plasmid pIgTAWETGQA followed by a booster immunization with AdTAWETGQA. We consider this approach complementary to the one described above because plasmid or adenovirus may used distinct routes for stimulating CD8^+^ T cells.

**Figure 5:**
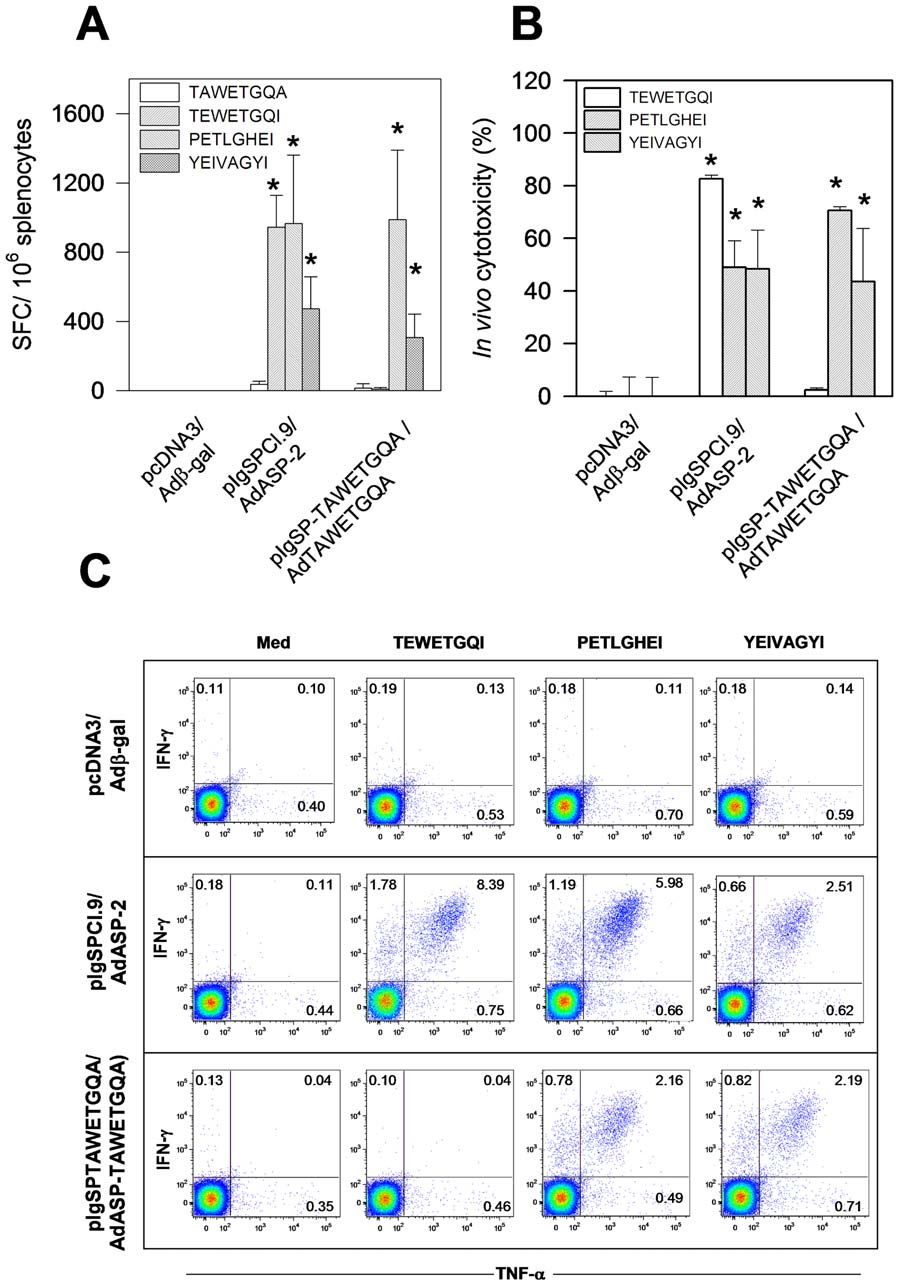
CD8 immune responses in A/Sn mice immunized with *asp-2* using the heterologous DNA prime-adenovirus boost vaccination regimen. A/Sn mice were primed i.m. with 100 µg of plasmids pcDNA3, pIgSPCl.9 or pIgSPTAWETGQA. Three weeks later, these mice were boosted i.m. with 2×10^8^ pfu Adβ-gal, AdASP-2 or AdTAWETGQA. A- Two weeks after the last dose, splenic cells were re-stimulated *in vitro* in the presence of medium only or the indicated peptides at a final concentration of 10 µM. The number of splenic IFN-γ spot forming cells (SFC) was estimated by *ex vivo* ELISPOT assay. B- *In vivo* cytotoxic activity was estimated by injecting each mouse with syngeneic CFSE-labeled splenic cells coated with or without 2 µM of the indicated peptide. Results are expressed as mean ± SD of 4 mice per group and are representative of experiments performed at least twice with similar results. Asterisks denote that the number of SFC or *in vivo* cytotoxicity were significantly higher when compared to SFC found in naïve or pcDNA3/Adβ-gal injected mice (*P*<0.01). C- Fourteen days after the last dose, these mice had their splenic cells cultured in the presence of anti-CD28 and Medium or the indicated peptides. After 12 h, cells were stained for CD8, IFN-γ and TNF-α. Examples of splenic CD8^+^ cells from immunized mice. Representative analyses (medians) are shown from four mice performed per experiment.

Immune responses were estimated 14 days after the booster immunization by ELISPOT following *ex vivo* stimulation with synthetic peptides encoding the immunodominant epitope TEWETGQI, the subdominant/cryptic epitopes PETLGHEI or YEIVAGYI or the mutated epitope TAWETGQA. We chose this protocol because the immune responses following heterologous prime boost immunization generates strong immune responses that can be easily detected after boosting [37]. We observed that mice immunized with *asp-2* genes (mutated or not) had specific IFN-γ producing cells when stimulated with peptides PELTHGEI or YEIVAGYI. The fact that these responses were of similar magnitude strongly argued that the expression/immunogenicity of both genes/antigens were very similar. We could detect IFN-γ producing cells specific for the TEWETGQI epitope only in mice immunized with pIgSPCl.9/AdASP-2. In contrast, none of the immunized mice presented IFN-γ producing cells when stimulated with the TAWETGQA peptide (Fig. 5A). The analysis of *in vivo* cytotoxic activity showed a similar picture (Fig. 5B). We also performed intra-cellular cytokine staining analysis for IFN-γ and TNF-α after *in vitro* peptide stimulation. As depicted in Fig. 5C, we detected CD8^+^ cells expressing IFN-γ and/or TNF-α specific for the TEWETGQI epitope only in mice immunized with pIgSPCl.9/AdASP-2 (Fig. 5C). Similar analyses performed 14 days after an infectious challenge with *T. cruzi* exhibited the same pattern of response (data not shown). Together, these immunological analyses demonstrated that heterologous prime-boost immunization with pIgSPTAWETGQA/AdTAEWTGQA failed to induce an immune response to the immunodominant epitope TEWETGQI but induced almost unaltered immune responses to the subdominant epitopes PETLGHEI or YEIVAGYI.

**Figure 6:**
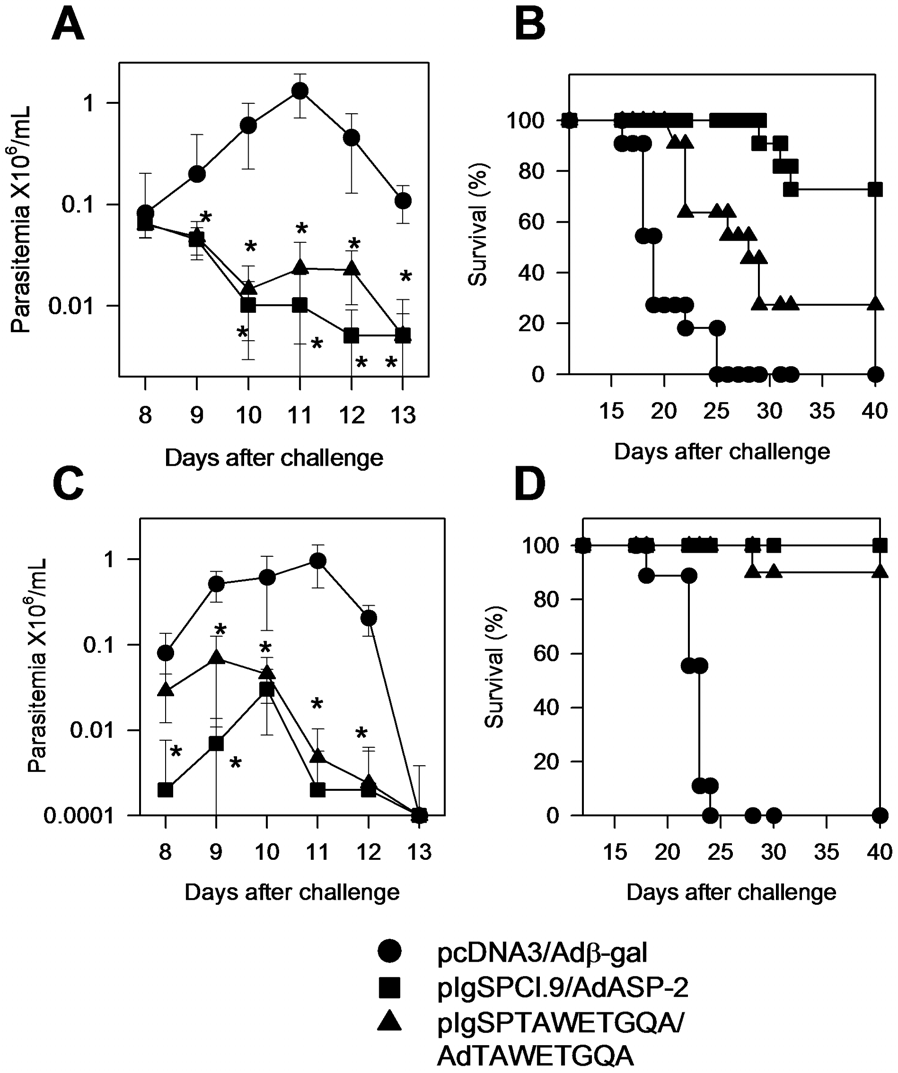
Trypomastigote-induced parasitemia and mortality in A/Sn mice immunized with *asp-2* using the heterologous DNA prime-adenovirus boost vaccination regimen. A/Sn mice were immunized as depicted in the legend of Fig. 5. Two weeks after the final immunizing dose, mice were challenged i.p. (Panels A and B) or s.c. (Panels C and D) with 150 bloodstream trypomastigotes. Parasitemia for each mouse group is represented as mean ± SD (n = 10 or 11). Asterisks denote that mice from groups immunized with pIgSPCl.9/AdASP-2 or pIgSPTAWETGQA/AdTAWTEGQA had significantly lower parasitemia (*P*<0.01) than pcDNA3/Adβ-gal-injected animals. Panels B and D represent Kaplan-Meier curves for survival of the mouse groups immunized and challenged as described above (n = 10 or 11). Mice immunized with pIgSPCl.9/AdASP-2 survived significantly longer than animals immunized with pIgSPTAWETGQA/AdTAWETGQA or pcDNA3/Adβ-gal (*P* = 0.01 or *P*<0.01, respectively). Mice immunized with pIgSPTAWETGQA/AdTAWETGQA survived significantly longer than pcDNA3/Adβ-gal-injected animals (*P*<0.01). Results are representative of two pooled experiments. No animals died after the 40^th^ day.

After challenge, parasitemia in mice immunized with the *asp-2* genes (mutated or not) was significantly lower than in control mice injected with pcDNA3/Adβ-gal (*P*<0.01, Fig. 6A). Although most of the mice immunized with pIgSPTAWETGQA/AdTAEWTGQA died after challenge, they survived longer than control mice injected with pcDNA3/Adβ-gal (Fig. 6B, *P*<0.01, LogRank test). In parallel, vaccinated and control mice were challenged by the s.c. route. We observed that the majority of the mice immunized with pIgSPTAWETGQA/AdTAEWTGQA survived the infectious challenge (Fig. 6D). We therefore concluded that broadening the T cell immune response using a genetic vaccination that elicits immune responses to subdominant/cryptic epitopes of ASP-2 provides protective immunity against infection. Nevertheless, likewise in the case of the plasmid DNA, after an i.p. challenge, in terms of survival, immunization with only genes expressing subdominant epitopes was not as effective as immunization with genes expressing both the dominant and the subdominant epitopes.

**Figure 7:**
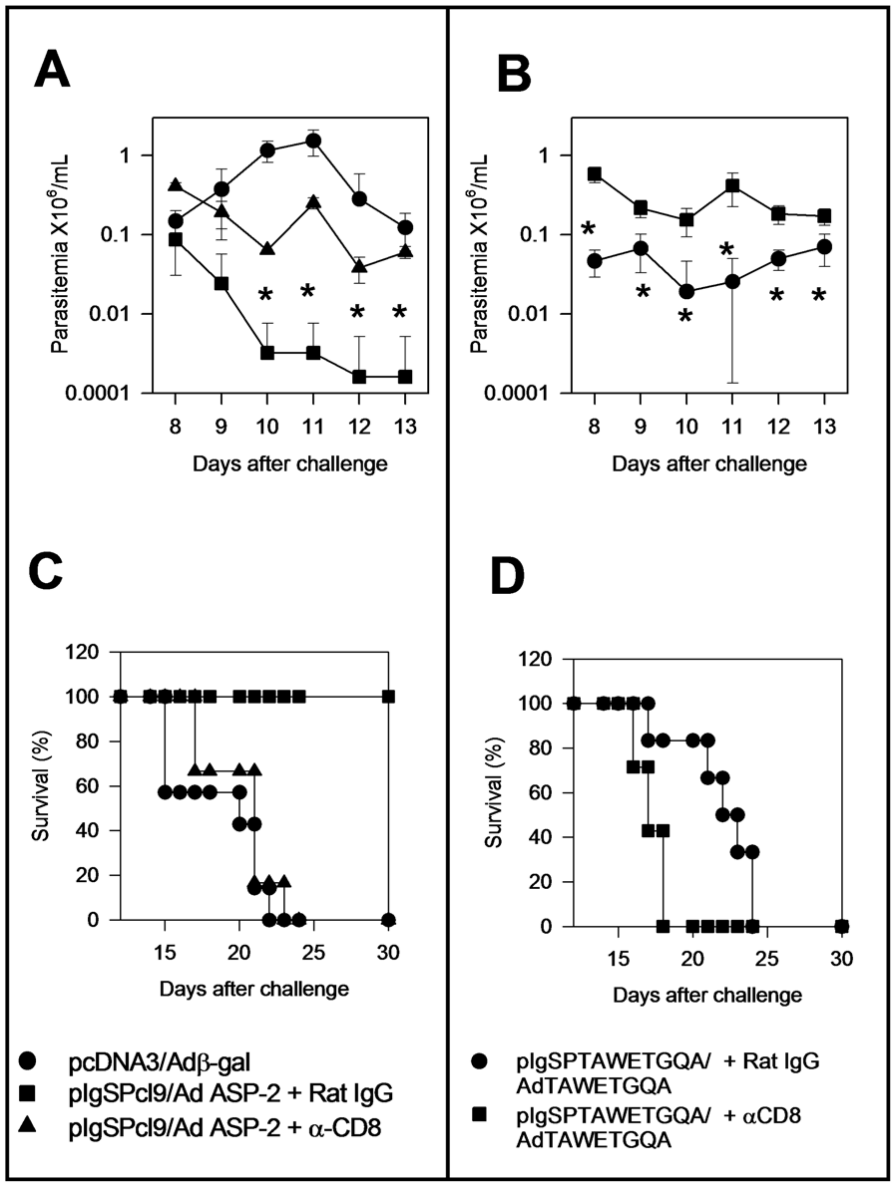
CD8 T cell dependence of protective immunity of A/Sn mice immunized with *asp-2* using the heterologous DNA prime-adenovirus boost vaccination regimen. A/Sn mice were immunized as described in the legend of Fig. 5. pIgSPTAWETGQA/AdTAWTEGQA had significantly lower parasitemia (*P*<0.01) than pcDNA3/Adβ-gal-injected animals. Before and after challenge, mice were treated as described in Methods section with rat IgG (control) or anti-CD8 MAb. The parasitemia for each mouse group is represented as mean ± SD (n = 6). Asterisks denote that mice from groups immunized with pIgSPCl.9/AdASP-2 or pIgSPTAWETGQA/AdTAWTEGQA and treated with Rat IgG had significantly lower parasitemia (*P*<0.01) than vaccinated mice treated with anti-CD8 (Panels A and B). Panels C and D represent Kaplan-Meier curves for survival of the mouse groups immunized and challenged as described above (n = 6). Mice immunized with pIgSPCl.9/AdASP-2 or pIgSPTAWETGQA/AdTAWETGQA and treated with Rat IgG survived significantly longer than vaccinated animals treated with anti-CD8 (*P*<0.01 in both cases).

Finally, to firmly establish that protective immunity was mediated by CD8^+^ T cells, we performed *in vivo* depletion experiments in mice vaccinated with the pIgSPCl.9/AdASP-2 or pIgSPTAWETGQA/AdTAEWTGQA. Treatment with anti-CD8 MAb renders these mice more susceptible to infection. CD8 depleted mice presented higher parasitemia (Fig. 7A and B) and shorter survival times (Fig. 7C and D) when compared to A/Sn mice vaccinated with heterologous prime-boost regimen (pIgSPCl.9/AdASP-2 or pIgSPTAWETGQA/AdTAEWTGQA) and treated with rat IgG. CD8 depleted mice had their survival time reduced to the same time as control mice which were injected with pcDNA3/Adβ-gal.

## Discussion

Here, we initially confirmed and extended our previous observation that experimental infection with the human intracellular pathogen *T. cruzi* restricted the repertoire of CD8^+^ T cells. While immune cells of infected H-2^a^ mice recognized a single immunodominant epitope of ASP-2, cells from mice immunized with recombinant genetic vaccines expressing this same *T. cruzi* antigen recognized, in addition to the immunodominant epitope, two other subdominant/cryptic epitopes. The sub-dominant epitopes not only failed to elicit IFN-γ and *in vivo* cytotoxicity during infection (Fig. 2), but they also did not stimulate TNF-α, IL-2 or IL-10 secretion by CD8^+^ T cells (Fig. 3). The precise reason for this strong immunodominant pattern during *T. cruzi* infection is unknown at present. One possible explanation for this biased immune response could be to provide an advantage to the parasite by avoiding an even higher and broader immune response. This hypothesis is in agreement with earlier studies showing that immunity to subdominant epitopes can provide an important contribution to protective immunity against viral infection [34], [40]–[43]. Our results also corroborated this hypothesis. Immunity to epitopes that are not commonly recognized during infection (cryptic) provided a significant degree of protective immunity. Nonetheless, immune responses against these cryptic epitopes did not substitute completely for CD8^+^ T cells specific for the immunodominant TEWETGQI epitope. We observed that immunization with plasmids alone or in combination with a recombinant adenovirus that did not express the immunodominant epitope elicited immune responses to the subdominant/cryptic epitopes but failed to provide optimal protective immunity when compared to a plasmid that expresses both the immunodominant and subdominants epitopes (Fig. 4D, 6B and 7D). These results demonstrate that the response to the immunodominant epitope contributes to the immunity elicited by genetic vaccination and is might be required for highly efficient resistance.

In previous studies, we showed that immunization with short proteins in the presence of the TLR9 agonist CpG elicited CD8^+^ T-cell mediated immunity against *T. cruzi* infection in A/Sn mice [44]. These short proteins contained only the AA 261 to 500 or 261 to 380. In both cases, they did not express the subdominant/cryptic epitopes. Based on that, we concluded that immunization with the immundominant CD8 epitope alone could provide a high degree of protective immunity even in the absence of the subdominant epitopes [44]. The fact that the immune response directed solely to the immunodominant epitope can provide significant degree of protective immunity against protozoan parasites has been established a long time ago by the use of adoptively transferred T cell clones or heterelogous-prime boost vaccination regimen [45]–[49].

In earlier studies in which we depleted CD8^+^ T cells from genetically vaccinated mice, we observed that these mice were unable to control parasitemia and died at the same time as control unvaccinated animals [37]. Therefore, although genetic immunization elicits effector CD4^+^ T cells, these cells do not account for the protection we observed. This concept was further corroborated with experiments of CD8 T cell-depletion performed here (Fig. 7A to D).

Using a different approach, a similar conclusion was also reached by Rosemberg *et al*., 2010 [17]. In their study, they induced simultaneous tolerance to two immunodominant *T. cruzi* epitopes in a resistant mouse strain. Following infection, an increased susceptibility to infection was observed. Nevertheless, they were still able to control and survive the experimental infection. This protective immunity was possibly mediated by CD8^+^ T cells specific to subdominant epitopes that substituted for the immunodominant ones. The AA sequences of these subdominant/cryptic epitopes have yet to be identified. Together with our study, they strongly support the notion that the immune responses to both dominant and subdominant/cryptic epitopes can be important for controlling experimental *T. cruzi* infection in inbred mouse strains. These results are in agreement with the observation with other parasites such as *Plasmodium*. Tolerance to or removal of the the immunodominant CD8 epitope of *P. yoelii* led to the development of immunity to CD8 subdominant epitopes as well [50], [51].

The mechanism operating during *T. cruzi* infection to restrict the immune response leading to immunodominance has yet to be characterized. We provide initial evidence that it could be explained by T cell competition for APCs by showing that in mice infected simultaneously with two different parasite strains containing different immunodominant epitopes, we could generate maximal responses to both epitopes without immunodominance or competition [15]. Our interpretation was that if the epitopes are presented by different APCs, then the immunodominant pattern is disrupted. However, a more formal demonstration using bone marrow chimeric mice studies is lacking. At the molecular level, this strong immunodominance can be explained by the type of antigen presentation that predominates during *T. cruzi* infection. In recent studies, important evidence has been provided that subdominant epitopes can only be directly presented by the expressing cells, which might occur in the case of the recombinant adenovirus. However, during indirect priming (cross-priming), these epitopes would be at a disadvantage [52]. *T. cruzi* may use cross-priming as the dominant route, drastically reducing the priming of subdominant epitopes.

In addition to shedding some light on the host-parasite relationship, our results may have important implications for the development of T cell vaccines against parasitic diseases. In our earlier studies, we observed that genetic vaccination with a heterologous prime-boost regimen employing plasmid DNA and recombinant adenovirus elicited strong, long lasting CD8^+^ T cell-mediated protective immunity against experimental infection in a mouse strain highly susceptible to *T. cruzi* infection [37], [38]. Here, we demonstrated that the CD8 T cell-mediated protective immunity observed was directed to three distinct epitopes, two of which are cryptic. The strategy of redirecting immunity to epitopes that are not usually targets of the naturally acquired immune response has been proposed as a possible means to improve immunity against viral infection [40]–[43]. Recently, this strategy was also proven useful to improve vaccination against *T. cruzi* infection [53].

Finally, our observations may have important implications regarding the basis for the strong immunodominance pattern observed after experimental infection with other intracellular parasites such as *Plasmodium*, *Toxoplasma gondii* and *Theileria parva* [50], [51], [54]-[57].
